# The Extract of *Aster Koraiensis* Prevents Retinal Pericyte Apoptosis in Diabetic Rats and Its Active Compound, Chlorogenic Acid Inhibits AGE Formation and AGE/RAGE Interaction

**DOI:** 10.3390/nu8090585

**Published:** 2016-09-21

**Authors:** Junghyun Kim, Kyuhyung Jo, Ik-Soo Lee, Chan-Sik Kim, Jin Sook Kim

**Affiliations:** Korean Medicine Convergence Research Division, Korea Institute of Oriental Medicine, Daejeon 34054, Korea; dvmhyun@kiom.re.kr (J.K.); jopd7414@kiom.re.kr (K.J.); knifer48@kiom.re.kr (I.-S.L.)

**Keywords:** advanced glycation end products, diabetic retinopathy, *Aster koraiensis*

## Abstract

Retinal capillary cell loss is a hallmark of early diabetic retinal changes. Advanced glycation end products (AGEs) are believed to contribute to retinal microvascular cell loss in diabetic retinopathy. In this study, the protective effects of *Aster koraiensis* extract (AKE) against damage to retinal vascular cells were investigated in streptozotocin (STZ)-induced diabetic rats. To examine this issue further, AGE accumulation, nuclear factor-kappaB (NF-κB) and inducible nitric oxide synthase (iNOS) were investigated using retinal trypsin digests from streptozotocin-induced diabetic rats. In the diabetic rats, TUNEL (Terminal deoxynucleotidyl transferase mediated dUTP Nick End Labeling)-positive retinal microvascular cells were markedly increased. Immunohistochemical studies revealed that AGEs were accumulated within the retinal microvascular cells, and this accumulation paralleled the activation of NF-κB and the expression of iNOS in the diabetic rats. However, AKE prevented retinal microvascular cell apoptosis through the inhibition of AGE accumulation and NF-κB activation. Moreover, to determine the active compounds of AKE, two major compounds, chlorogenic acid and 3,5-di-*O*-caffeoylquinic acid, were tested in an in vitro assay. Among these compounds, chlorogenic acid significantly reduced AGE formation as well as AGE/RAGE (receptor for AGEs) binding activity. These results suggest that AKE, particularly chlorogenic acid, is useful in inhibiting AGE accumulation in retinal vessels and exerts a preventive effect against the injuries of diabetic retinal vascular cells.

## 1. Introduction

Retinal vascular cells undergo cell death and functional disorders in chronic hyperglycemia [1]. Retinal pericyte loss is an early sign of diabetic retinopathy and elicits the formation of microaneurysms, neovascularization and vascular hemorrhages. Retinal cell death causes permanent vision loss. 

Advanced glycation end products (AGEs) are chemically heterogeneous and sugar-derived irreversible protein adducts that are known to be associated with the progression of diabetic vasculopathy [2,3]. AGEs are also believed to be one of the major factors that contribute to diabetic retinopathy. Indeed, the levels of AGEs are much higher in diabetic patients [2]. Increased AGE accumulation is closely associated with the severity of diabetic retinopathy [4,5]. Moreover, the administration of exogenous AGEs induces diabetes-like microvascular changes, such as the loss of pericytes [6]. AGE-induced apoptosis has been reported to be mediated via pro-apoptotic cytokines or the increased oxidative stress induced by AGEs and the interactions of receptors for AGEs (RAGEs) [7,8,9]. 

Nitric oxide (NO) is one of the important substances that regulate various physiological and pathological systems, including vasodilation, neurotransmission and cell survival. NO is produced from l-arginine as the substrate of several forms of NO synthase (NOS). NOS1 (neuronal NOS, nNOS) and NOS3 (endothelial NOS, eNOS) are constitutively expressed. NOS2 (inducible NOS, iNOS) is usually not expressed in normal resting cells but is induced by various cytokines and endotoxins [10]. The induction of iNOS generates large quantities of NO that could be harmful to surrounding cells [11,12]. NO is also a well-known mediator of apoptosis, and its effect can be anti-apoptotic or pro-apoptotic [13]. Previous evidence has demonstrated that upregulations of iNOS levels have been found in retinal cells of human patients and experimental diabetic animals [14,15,16,17]. Diabetic mice with genetic disruptions of iNOS exhibit reduced alteration of retinal capillaries [18]. These findings suggest that an elevated production of NO is closely correlated with the risk of developing diabetic retinopathy. AGEs have recently been found to stimulate the activation of nuclear factor-kappaB (NF-κB) through their interactions with RAGE [19]. NF-κB activation is known to induce elevations in iNOS levels because the promoter of iNOS has NF-κB binding site mRNA [20,21,22,23,24]. Thus, it is possible that AGEs induce the activation of both NF-κB and iNOS. Therefore, based on these results, three potential therapeutic targets for diabetic retinopathy have been proposed: (1) suppression of AGE formation; (2) blockade of the AGE/RAGE interaction; and (3) inhibition of the AGE/RAGE-mediated down-stream signaling pathways.

*Aster koraiensis* is commonly called Korean starwort and is a valuable, perennial, Korean native plant. This taxon is discriminated from other related genera, such as *Aster, Heteropappus*, and *Kalimeris,* by its achene without pappi [25]. In Korea, the young leaves of *Aster koraiensis* are used in food preparation, and the roots have been traditionally used as foodstuffs or medicinal materials without any scientific proof. The herbs have typically been used as appetizers, side dishes, and folk medicine to control various diseases, such as pneumonia, chronic bronchitis and pertussis [26,27]. However, the pharmacological activities of *A. koraiensis* that are relevant to diabetic retinopathy are undetermined. In the present study, we investigated the therapeutic effects of *A. koraiensis* extract (AKE) on the degeneration of diabetic retinal vascular cells in STZ-induced diabetic rats. We further investigated the inhibitory effects of chlorogenic acid and 3,5-di-*O*-caffeoylquinic acid from *A. koraiensis* on AGE formation, the AGE/RAGE interaction and NF-κB activation.

## 2. Materials and Methods

### 2.1. Plant Material and Preparation of AKE

The aerial and root parts of *A. koraiensis,* were purchased from Gongju, Chungchengnamdo, South Korea in August 2007 and 2009, respectively. The used aerial parts*,* including the flowers, leaves and stems (AKE) was prepared according to a previously reported method [28]. Briefly, the dried and ground plant material (2.5 kg) was extracted with EtOH (3 × 20 L) by maceration at room temperature for 3 days. The extracts were combined and concentrated in vacuo at 40 °C to give an EtOH extract (303 g). A voucher specimen (No. KIOM-83A) was deposited for future reference (Herbarium No. KIOM-83A). 

### 2.2. Isolation of 3,5-di-O-Caffeoylguinic Acid and Chlorogenic Acid 

The 3,5-di-*O*-caffeoylguinic acid was isolated from AKE as previously described [29]. The EtOH extract was dissolved in water and continuously partitioned with solvents, then concentrated to give extracts of *n*-hexane (37.5 g), EtOAc (34.4 g), *n*-BuOH (81.3 g), and water (149 g) layer, respectively. The EtOAc extract (34.4 g) was chromatographed on a Silica gel column (CHCl_3_/EtOAc = 20:1→2:1, CHCl_3_/MeOH = 3:1→1:1, CHCl_3_/MeOH/Water = 7:4:1) to afford ten fractions (F01-F10). Chromatographic separation of fraction F09 (8.56 g) was carried out by MPLC (medium pressure liquid chromatography) using isocratic solvent systems consisting of water and MeOH (water: 90%, 60 min; 80%, 90 min; 70%, 60 min; 60%, 70 min; MeOH: 100%; column: Ultra Pack C; flow rate: 12 mL/min) to produce sixteen subfractions (F0901-F0916). 3,5-di-*O*-caffeoylguinic acid (white powder, 170 mg) purified by precipitation in MeOH from the subfraction F0904. Chlorogenic acid was isolated from the root of *A. koraiensis* as follows. The dried roots of *A. koraiensis* (2.8 kg) were extracted with EtOH. The EtOH extract was dissolved in water and continuously partitioned with solvents, then concentrated to give extracts of *n*-hexane (7 g), EtOAc (40.0 g), and an aqueous residue. The EtOAc extract (40 g) was chromatographed over a silica gel column chromatography using a CHCl_3_/MeOH gradient system (1:0→0:1 v/v) to yield 11 fractions (F01-F11). F10 (4.45 g) was further purified over Sephadex LH-20 eluted with MeOH/H_2_O gradient (1:0→0:1 v/v) to afford chlorogenic acid (3-*O*-caffeoylquinic acid, 50 mg).

### 2.3. High Performance Liquid Chromatography Analysis

**Figure 1:**
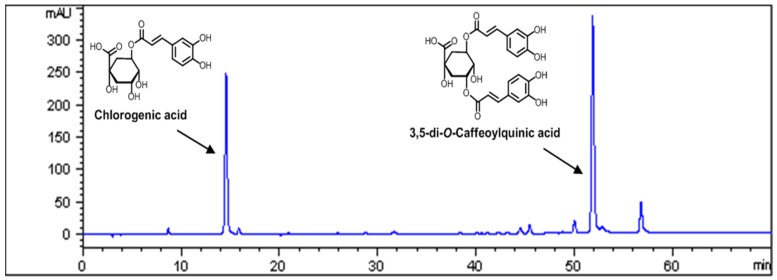
The HPLC (high performance liquid chromatography) chromatogram of the EtOH (ethanol) extract of *Aster koraiensis* (AKE) with UV (ultraviolet) detection at 325 nm.

The contents of major compounds in AKE were determined by high performance liquid chromatography (HPLC) analysis. The peaks of chlorogenic acid and 3,5-di-*O*-caffeoylquinic acid were confirmed from standard chemicals (Sigma Aldrich, St. Louis, MO, USA). A Runa C-18 analytical column (i.d., 4.6 mm × 250, 5.0 µm, Phenomenex, Torrance, CA, USA) was used with the mobile phase consisting of 0.1% acetic acid in water (A) and acetonitrile (B). The mobile phase gradient elution was programmed as follows: 0–25 min, 90%–86% A; 25–60 min, 86%–75% A; 60–65 min, 75% A. The flow rate of the mobile phase was set at 1.0 mL/min. The column temperature was maintained at 30 °C. The sample injection volume was set at 5 µL and a MWD (multiple wavelength detector) set at 325 nm (Figure 1). 

### 2.4. AGE Formation Assay 

Seven milligrams of bovine serum albumin (BSA; Sigma, St. Louis, MO, USA) in 700 μL of 50 μM phosphate buffer (pH 7.4) were added to 100 μL of 200 mM glucose or fructose and then mixed with 200 μL of various concentrations of the test compounds or aminoguanidine (AG, Sigma). After 14 days of incubation at 37 °C, the fluorescent products of glycated-BSA was determined using a spectrofluorometer (Ex: 350, Em: 450 nm; Synergy HT, BIO-TEK, Winooski, VT, USA). Each experiment was repeated three times. The 50% inhibition concentration (IC_50_) for AGE formation was calculated by interpolation from the concentration-inhibition curve.

### 2.5. AGE/RAGE Binding Activity Assay

AGE-BSA was purchased from Wako Pure Chemical Industries, Ltd. (Osaka, Japan) and labeled with horseradish peroxidase using a kit (Peroxidase Labeling Kit-NH_2_, Dojindo Molecular Technologies, Inc., Tokyo, Japan). The interaction between AGE and RAGE was assayed using a protocol reported earlier [30,31,32]. Briefly, AGE-BSA and the test compounds were added to RAGE-coated, 96-well plates. The binding of the AGE-BSA to RAGE was determined using a 3,3′,5,5′-tetramethylbenzidine enzyme-linked immunosorbent assays (TMB-ELISA) substrate solution (Sigma). The inhibition of the AGE/RAGE interaction is expressed as the percentage of the binding of the AGE-BSA with RAGE in the presence of the test compounds.

### 2.6. Animals and Induction of Diabetes

All experiments were conducted in accordance with the National Institutes of Health (NIH) Guide for the Care and Use of Laboratory Animals and the ARVO Statement for the Use of Animals in Ophthalmic and Vision Research The experimental protocol was approved by the Institutional Animal Care and Use Committee (No. L08010-16852). Hyperglycemia was induced by an intraperitoneal administration of 60 mg/kg streptozotocin (STZ, Sigma) in 7-week-old male Sprague Dawley (SD) rats (Orient Bio, Seoul, Korea). Age-matched normal rats were administered a vehicle (10 mM citrate buffer, pH 4.5). One week after STZ injection, hyperglycemic rats (defined by a glucose concentration of greater than 300 mg/dL) and normoglycemic rats were divided into three groups: (1) a normal control group (*n* = 8) and hyperglycemic rats were divided into two groups (2) a diabetic group (*n* = 8); and (3) an AKE-treated group (100 mg/kg body weight, *n* = 8). AKE extract was administered once per day orally for 4 months. 

### 2.7. Trypsin-Digested Vessel Preparation

At necropsy, each eye was enucleated and then fixed with 4% paraformaldehyde for 24 h. Each retina was isolated under a dissecting microscope and entirely washed with copious amounts of water. The retinas were incubated in 3% trypsin in sodium phosphate buffer for 1 h. Following the completion of digestion, the isolated retinal vasculatures were stained with periodic acid–Schiff–hematoxylin (PAS) and mounted.

### 2.8. Terminal Deoxynucleotidyl Transferase Mediated dUTP Nick End Labeling (TUNEL) Staining

The retinal vasculature was stained with a DeadEnd apoptosis detection system (Promega, Madison, WI, USA). TUNEL-positive cells were labeled with fluorescein-conjugated streptavidin. The numbers of TUNEL-positive cells were determined per unit area (mm^2^).

### 2.9. Immunohistochemical Staining

Immunohistochemical staining was performed according to previously reported protocols [33]. The antibodies used in this study were mouse anti-AGEs (6D12, Cosmo bio, Tokyo, Japan), rabbit anti-iNOS (Cell Signaling, Denver, MA, USA), rabbit anti-cleaved caspase-3 (Cell Signaling) and mouse anti-NF-κB (MAB3026, Chemicon International, Temecula, CA, USA). To detect AGEs, iNOS and NF-κB, the slides were labeled with a LSAB kit (DAKO, Carpinteria, CA, USA) and visualized with a DAB substrate kit (DAKO). For the detection of cleaved caspase-3, the sections were incubated with tetramethylrhodamine-conjugated goat anti-rabbit IgG (Santa Cruz Biotechnology, Santa Cruz, CA, USA) and detected by fluorescence microscopy (BX51, Olympus, Tokyo, Japan). For the morphometric analyses, the immunoreactive intensity per unit area (mm^2^) was measured using an ImageJ software (NIH, Bethesda, MD, USA).

### 2.10. In Situ Hybridization 

The oligoprobe for iNOS for the in situ hybridization was synthesized as previously reported [34], and the antisense sequences for rat iNOS mRNA was 5′-CGTCATTTCTTCCTGATAGAGGTG GTCGTCCTCCTCTGGGTGCCTGCATGAGGAAGTATGAGGGGCCAAAAGGAAAGAGAATGTG-3′. The corresponding sense oligoprobe was used for the negative controls. The sense and antisense oligoprobes were tagged with digoxigenin using a kit (DIG Oligonucleotide Tailing Kit, Roche Diagnostics, Mannheim, Germany). We performed in situ hybridization according to protocols that have previously been reported [35]. For the quantitative analyses, the positive signal intensity per unit area (mm^2^) was calculated using ImageJ software (NIH).

### 2.11. Statistical Analysis

Significant differences between groups were analyzed using one-way analysis of variance (ANOVA) and *Tukey's multiple comparison* test in the Prism 5.0 software (GraphPad, San Diego, CA, USA), and *p* < 0.05 was considered statistically significant.

## 3. Results

### 3.1. Standardization of AKE

Quality control of herbal medicines is difficult because the materials are derived from many different sources. Therefore, to assure the quality of the AKE, we performed HPLC analyses. Chlorogenic acid and 3,5-di-*O*-caffeoylquinic acid were found to be the major components of AKE (Figure 1), and the contents of these components in AKE were 1.24% and 2.25%, respectively (Table 1). 

### 3.2. Inhibitory Effects of 3,5-di-O-Caffeoylquinic Acid and Chlorogenic Acid on AGE Formation in Vitro

Chlorogenic acid and 3,5-di-*O*-caffeoylquinic acid were subjected to in vitro bioassays to evaluate the formation of AGE-BSA. The inhibitory effects of 3,5-di-*O*-caffeoylquinic acid and chlorogenic acid on the formation of AGE-BSA are summarized in Table 2. In this study, 3,5-di-*O*-caffeoylquinic acid and chlorogenic acid exhibited much stronger inhibitory activities on AGE-BSA formation (IC_50_ values of 148.32 ± 3.14 and 6.59 ± 0.04 μM, respectively) than the well-known glycation inhibitor aminoguanidine (IC_50_ value of 962 ± 29.52 μM). The IC_50_ value of 3,5-di-*O*-caffeoylquinic acid was also previously reported [29].

### 3.3. Inhibitory Effects of AKE, 3,5-di-O-Caffeoylquinic Acid and Chlorogenic Acid on AGE/RAGE Binding Activity in Vitro

**Figure 2:**
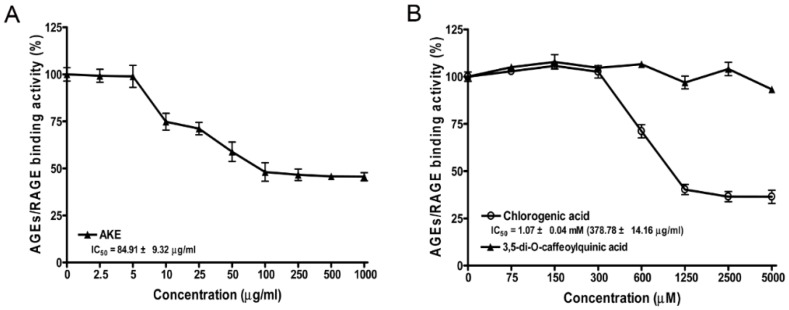
Advanced glycation end products/receptor for AGE (AGE/RAGE) binding activity of AKE (**A**); Chlorogenic acid and 3,5-di-*O*-caffeoylquinic acid (**B**). The percentages of AGE/RAGE binding activities were calculated relative to the vehicle control (0 concentration). The IC_50_ values were calculated from the dose inhibition curves. All data are presented as the mean ± the SD (*n* = 4).

The inhibitions of AGE-BSA binding to RAGE at various concentrations of 3,5-di-*O*-caffeoylquinic acid and chlorogenic acid were tested (Figure 2). In this assay, AKE and chlorogenic acid and dose-dependently markedly reduced AGE-BSA/RAGE binding activity, and the IC_50_ values of AKE and chlorogenic acid were 84.91 ± 9.32 and 378.78 ± 14.16 μg/mL, respectively. However, 3,5-di-*O*-caffeoylquinic acid did not affect AGE/RAGE binding activity or the binding of AGE–BSA and RAGE. 

### 3.4. Body Weight and Blood Glucose

As indicated in Table 3, all of the STZ-induced diabetic rats exhibited significantly elevated blood glucose levels and reduced body weights compared with the normal control rats (*p* < 0.01). However, treatment with AKE did not show any significant change on hyperglycemia or body weight compared to the vehicle-treated diabetic rats.

### 3.5. Apoptosis of Retinal Microvascular Cells

**Figure 3:**
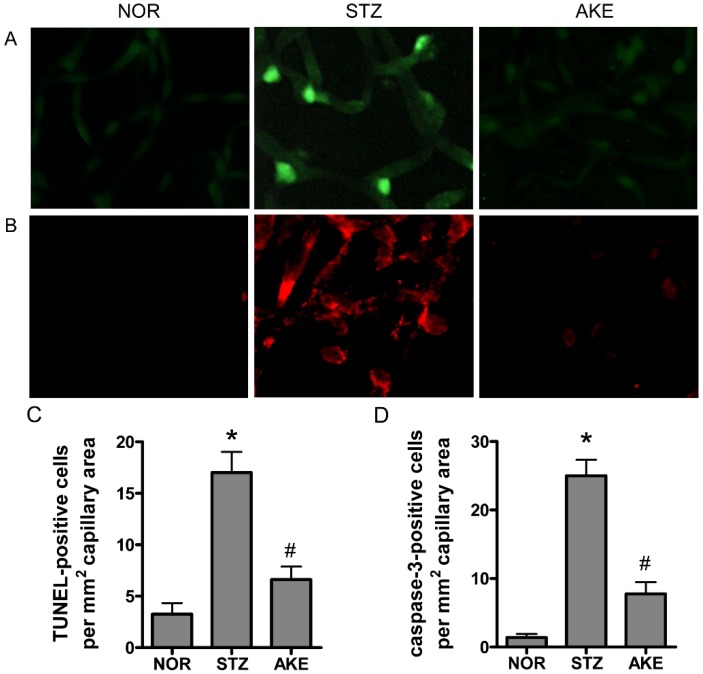
Apoptosis in the retinal capillaries. The trypsin-digested retinas from normal rats (NOR), STZ-induced diabetic rats (STZ) and AKE-treated diabetic rats (AKE) were stained with (**A**) Terminal deoxynucleotidyl transferase mediated dUTP Nick End Labeling (TUNEL; green) and (**B**) cleaved caspase-3 (red). 400× magnification. Quantitative analysis of (**C**) TUNEL-positive nuclei and (**D**) caspase-3-positive cells. All data are expressed as the mean ± the standard error of the mean (SEM), *n* = 8. * *p* < 0.01 vs. normal rats, ^#^ *p* < 0.01 vs. STZ-induced diabetic rats.

To characterize the death of retinal vascular cells, we applied the TUNEL assay and cleaved caspase-3 immunostaining. In the retinal trypsin digests of normal rats, barely any TUNEL-positive cells were observed. In the diabetic group, many TUNEL-positive microvascular cells and numerous fragmented nuclei were observed in the retinal trypsin digests (Figure 3A). Similarly, enhanced caspase-3-positive cells were observed in the retinal capillaries of the diabetic rats (Figure 3B). However, the treatment with AKE in the diabetic rats prevented the increase in positive cells and resulted in a level similar to that observed in the normal rats (Figure 3C,D).

### 3.6. AGE Staining of the Retinal Microvascular Cells

**Figure 4:**
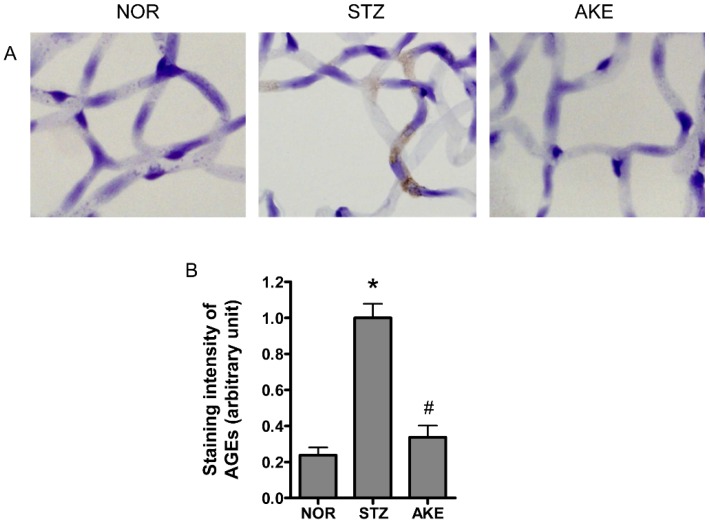
Immunohistochemical localization of the advanced glycation end products (AGEs). (**A**) Representative immunostaining of AGEs in the retinal capillaries of normal rats (NOR), STZ-treated diabetic rats (STZ) and AKE-treated diabetic rats (AKE). Strong immunoreactivity for AGEs was observed in the cytoplasm of the diabetic retinal microvascular cells. In contrast, immunoreactivity in the capillary cells of the AKE-treated diabetic rats was decreased. 400× magnification; (**B**) Quantitative analysis of the AGEs immunoreactive intensities. The values in the bar graphs represent the means ± the SEM, *n* = 8. * *p* < 0.01 vs. normal rats, ^#^ *p* < 0.01 vs. STZ-induced diabetic rats.

To investigate AGE formation and accumulation in the retinal microvascular cells, immunohistochemical staining for AGEs was performed on the retinal trypsin digests. In the normal rats, only weak staining for AGEs was present within the retinal microvascular cells. In the diabetic rats, a striking increase in immunoreactive straining for AGEs was observed in the cytoplasm of the retinal microvascular cells (Figure 4A). The treatment of the diabetic rats with AKE reduced the AGEs deposited in the retinal microvascular cells. Morphometric analysis revealed that the intensity of AGE immunolabeling was significantly increased by 5-fold in diabetic rats compared with the normal rats (Figure 4B, *p* < 0.01), and this increase was suppressed by treatment with AKE (Figure 4B, *p* < 0.01).

### 3.7. Expression of iNOS mRNA and iNOS Protein

**Figure 5:**
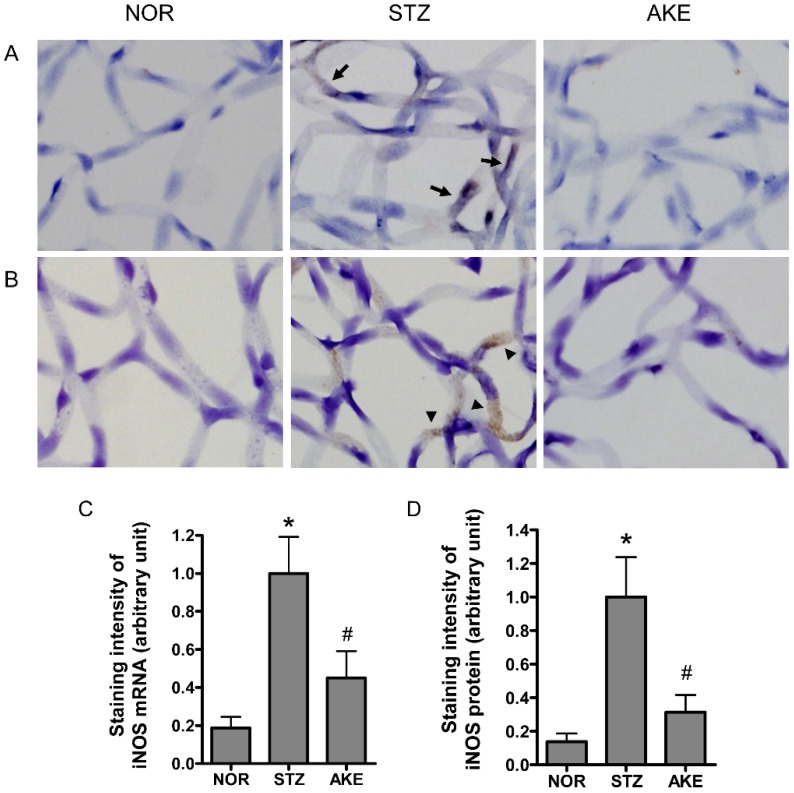
The expression pattern of inducible NOS (iNOS). (**A**) Distribution of iNOS mRNA as detected by in situ hybridization. The diabetic retinal microvascular cells exhibited strong hybridization signal for iNOS (arrow). The AKE-treated capillary cells rarely exhibited signals compared to the diabetic cells; (**B**) Immunohistochemical localization of the iNOS protein. iNOS immunoreactivity (arrowhead) was observed in the cytoplasm of the diabetic retinal microvascular cells. The immunoreactivity in the AKE-treated retinal microvascular cells was decreased in intensity. NOR, normal rats; STZ, STZ-induced diabetic rats; AKE, AKE-treated diabetic rats. 400× magnification; Quantitative analysis of (**C**) iNOS mRNA and (**D**) iNOS protein signal intensities. The values in the bar graphs represent the mean ± the SEM, *n* = 8. * *p* < 0.01 vs. normal rats, ^#^ *p* < 0.01 vs. STZ-induced diabetic rats.

The localization of iNOS mRNA was detected by in situ hybridization. In the retinal trypsin digests of the diabetic rats, iNOS mRNA was expressed at markedly higher levels in the cytoplasm of the microvascular cells than in the digests of the normal rats (Figure 5A). Similarly, the iNOS protein was highly expressed in the cytoplasm of the retinal microvascular cells of the diabetic rats as assessed by immunohistochemistry (Figure 5B). However, the treatment with AKE markedly reduced the expression level of iNOS mRNA. Similarly, a remarkable reduction in the level of iNOS protein was observed in the AKE-treated diabetic retinal vessels (Figure 5C,D).

### 3.8. Activation of NF-κB in the Retinal Microvascular Cells 

**Figure 6:**
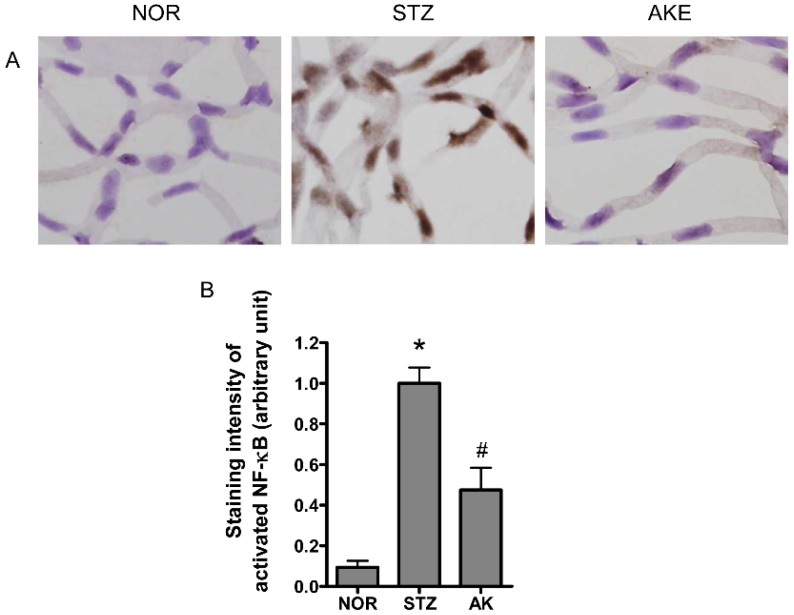
Distribution of activated nuclear factor-kappaB (NF-κB) in the retinal microvascular cells detected by immunohistochemistry. (**A**) Representative photomicrographs of retinal microvasculatures from normal rats (NOR), STZ-induced diabetic rats (STZ) and AKE-treated diabetic rats (AKE). Positive signals (arrow) for activated NF-κB were mainly detected in the nuclei of the diabetic retinal microvascular cells. 400× magnification; (**B**) Quantitative analysis of the positive cells in the retinal microvascular. The values in the bar graphs represent the mean ± the SEM, *n* = 8. * *p* < 0.01 vs. normal rats, ^#^ *p* < 0.01 vs. STZ-induced diabetic rats.

The transcription factor NF-κB is a common component of the downstream signal pathway of AGEs [36]. NF-κB also plays a key role in apoptosis [37,38]. Therefore, we evaluated the activation of NF-κB in the retinal microvascular cells with immunohistochemistry. Marked NF-κB activity was mainly found in the nuclei of the retinal microvascular cells of the diabetic rats (Figure 6A). However, in the normal rats, positive signals for activated NF-κB were rarely detected. AKE significantly inhibited the expression of activated NF-κB compared with the expression in diabetic rats (Figure 6B, *p* < 0.01).

## 4. Discussion

Several studies have demonstrated that retinal capillary cell apoptosis plays a critical role in the progression of the early stage of diabetic retinopathy [39,40]. In this study, we demonstrated that AKE exerted an inhibitory effect on the formation of AGEs and AGE/RAGE binding activity in vitro, and treatment with AKE prevented retinal microvascular cell apoptosis and reduced the formation of AGEs, NF-κB activation and iNOS expression in STZ-induced diabetic retinopathy. These results indicated that the oral administration of AKE resulted in a decline in the retinal microvascular damage mediated by AGEs, NF-κB and iNOS. 

The formation and accumulation of AGEs in the retina are closely associated with the risk of developing diabetic retinopathy [4,5]. Repeated intravenous injections of exogenous AGEs into normoglycemic animals leads to vascular basement membrane thickening in the retinas [41], the adhesion of leukocytes [42] and blood-retina barrier breakdown [43]. Recently, it was reported that AGEs induce retinal pericyte apoptosis due to pro-apoptotic cytokines or oxidative stress through the AGE/RAGE interaction [7,8,9]. Therefore, accumulating evidence suggests that the inhibition of AGE formation or the blockade of the interaction between AGEs and RAGE might represent pharmacological targets for the treatment of diabetic retinopathy [44]. Furthermore, it was recently reported that AGEs binding to the RAGE elicit the activation of NF-κB [45]. NF-κB activation leads to retinal pericyte loss [37] and apoptosis [38]. Moreover, activated NF-κB stimulates the expression of iNOS [20,21,22,23,24]. NO may serve as a cytotoxic effector molecule [46]. Increased expression of iNOS and excessive generation of NO have been detected in retinitis, uveitis, glaucoma and cataracts [47,48,49,50]. High levels of NO induce apoptosis in various cells [51,52,53,54]. It is generally known that iNOS generates relatively high concentrations of NO. Additionally, the vitreous level of NO is elevated in patients with diabetic retinopathy [55]. Furthermore, structural changes in retinal vessels are diminished in iNOS^−/−^ mice with diabetes [18]. In this study, we first demonstrated the retino-protective effect of AKE in diabetic animals. Pericyte loss induces alterations of retinal microvascular integrity [56]. AGEs can lead to apoptosis of retinal pericytes [1,57,58,59]. Our data regarding the upregulation of AGEs, NF-κB and iNOS in STZ-induced diabetic rats provide strong evidence that these factors are responsible for the retinal pericyte apoptosis. These results suggest that the anti-apoptotic effect of AKE is probably due to its inhibitory effect on AGEs, NF-κB and iNOS.

In a previous study, aminoguanidine was found to inhibit the development of diabetic retinopathy despite continued hyperglycemia [60]. AKE prevented retinal microvascular cell apoptosis through the inhibition of AGE accumulation, the inhibition of the AGE/RAGE interaction and components of its downstream signaling pathway, including NF-κB and iNOS, which independently affect hyperglycemia. In many cases, herbal medicines are known to exhibit synergistic effects of a variety of phytocompounds that are present in the herbs [61]. In the present study, we identified two major compounds (i.e., 3,5-di-*O*-caffeoylquinic acid and chlorogenic acid) in AKE. Each component exhibits protective activities against oxidative neuronal cell death [62,63,64]. Additionally, we clearly demonstrated that chlorogenic acid can significantly inhibit the formation of AGEs and the AGE/RAGE interaction in an in vitro assay, and 3,5-di-*O*-caffeoylquinic acid inhibited the formation of AGEs with approximately 20-fold greater efficacy than chlorogenic acid, which suggests that the anti-apoptotic activities of AKE may be ascribed to the synergistic effects of these two active components. 

Reactive carbonyl species (RCS) such as glyoxal, glyoxalaldehyde and methylglyoxal have been recognized as potent precursors of AGE formation [65]. Aminoguanidine can inhibit AGE accumulation by interacting with the highly reactive RCS and acting as a carbonyl scavenger [66]. In our previous study, we also showed that chlorogenic acid has a chelating activity of methyglyxoal [67]. Therefore, the chelating activity of chlorogenic acid can interfere with AGEs formation.

In the present study, an oral dose of 100 mg/kg AKE contained 1.24 mg/kg chlorogenic acid is recommended. Qi et al. reported that the peak plasma concentration of chlorogenic acid after oral administration of 50 mg/kg chlorogenic acid to rats was 550 μg/mL [68]. Although we used a relatively low dose of chlorogenic acid, a concentration of 52.55 μg/mL chlorogenic acid is sufficient to inhibit AGE formation in vitro. Thus, we chose an oral dose of 100 mg/kg AKE to inhibit retinal AGEs accumulations in the STZ-induced diabetic rats.

## 5. Conclusions

In conclusion, our study demonstrated that AGE accumulation in retinal vessels may enhance the expressions of NF-κB and iNOS, which lead to vascular cell apoptosis during the development of diabetic retinopathy. AKE has the ability to attenuate AGE accumulation, which results in the prevention of apoptosis. Taken together, our results suggest that treatment with AKE could likely be a valuable therapeutic approach to diabetic retinopathy.

## Figures and Tables

**Table 1:** The contents of chlorogenic acid and 3,5-di-*O*-caffeoylquinic acid in *Aster koraiensis* extract (AKE).

Compound	Content	
	mg/g	%
Chlorogenic acid	12.35 ± 0.22	1.24 ± 0.02
3,5-di-*O*-caffeoylquinic acid	22.54 ± 0.51	2.25 ± 0.05

All data are expressed as the mean ± the standard deviation (SD), *n* = 4.

**Table 2:** Inhibitory effects of chlorogenic acid and 3,5-di-*O*-caffeoylquinic acid in AKE on the formation of advanced glycation end products (AGE) in vitro ^a^.

Compound	Inhibitory Effect (IC_50_ Value) ^a^	
	μM	μg/mL
Chlorogenic acid	148.32 ± 3.14	52.55 ± 1.11
3,5-di-*O*-caffeoylquinic acid	6.59 ± 0.04	3.40 ± 0.02
Aminoguanidine ^b^	962 ± 29.52	71.19 ± 2.19

^a^ The concentration required for a compound to induce a 50% inhibition (IC_50_) of AGE formation. The IC_50_ values were calculated from the dose-inhibition curves. Inhibitory effects are expressed as the means of triplicate experiments; ^b^ Aminoguanidine was used as a positive control.

**Table 3:** Metabolic and physical parameters.

	NOR	STZ	AKE
Body weight (g)	494.46 ± 8.73	229.25 ± 10.66 *	233.51 ± 11.69 *
Blood glucose (mg/dL)	124.93 ± 10.8	409.78 ± 68.10 *	383.82 ± 131.71 *

NOR: normal rats; STZ: streptozotocin-induced diabetic rats; AKE: STZ-induced diabetic rats treated with AKE (100 mg/kg body weight). All data are expressed as the mean ± the SD. * *p* < 0.01 vs. normal rats.
